# Primary myxofibrosarcoma of the parotid: case report

**DOI:** 10.1186/1471-2407-10-246

**Published:** 2010-06-01

**Authors:** Xu Li, Xin Chen, Zhao-Hui Shi, Yang Chen, Jing Ye, Li Qiao, Jian-Hua Qiu

**Affiliations:** 1 Department of Otolaryngology-Head and Neck Surgery, Xijing Hospital, Fourth Military Medical University, Xi'an-710032, PR China; 2 Department of Pathology, Xijing Hospital, Fourth Military Medical University, Xi'an-710032, PR China

## Abstract

**Background:**

Myxofibrosarcoma is common in the extremities of elderly people and is characterized by a high frequency of local recurrence.

**Case presentation:**

We report a 37 year old female who presented with a 4-month history of facial pain and a 3-month history of painful progressive swelling in the preauricular area. She underwent a total parotidectomy. The tumor was histopathologically and immunohistochemically diagnosed as a low-grade myxofibrosarcoma. The patient was free of disease 9 months after surgery with uneventful post-operative clinical course.

**Conclusions:**

Parotid area swelling should always alert doctors. To our knowledge, this is the first case of parotid myxofibrosarcoma. It should be added to the differential diagnosis of diseases of the parotid. We have to recognize this disease and seek adequate treatment for it.

## Background

Myxofibrosarcoma is a fibroblast-derived sarcoma with character of myxoid areas in association with varying proportions of cellular areas showing a pleomorphic storiform pattern. It grows commonly in the subcutaneous of the extremities of elderly people, with rare occurrences in the head and neck (2.7%) [1].

Here we report a case of low-grade myxofibrosarcoma, arising in the parotid, and review the clinical, radiological and histopathological characteristics of this neoplasm. To our knowledge, such a presentation of parotid myxofibrosarcoma has not been reported before.

## Case presentation

**Figure 1:**
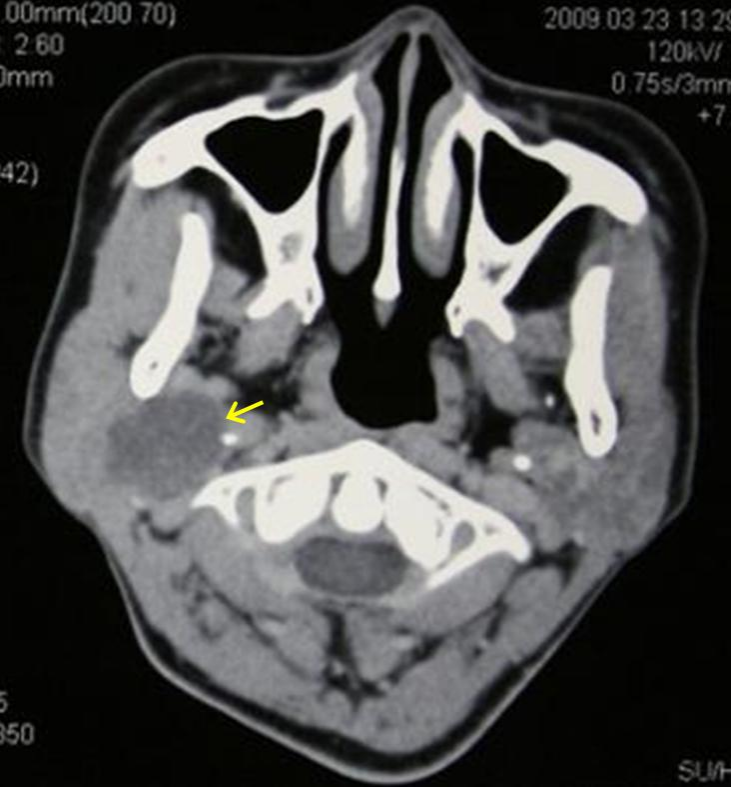
**CT scan of the patient**. A contrast enhanced Computed tomography (CT) scan revealed a well defined soft tissue mass (40 mm × 25 mm, arrow) in the right deep parotid lobe.

A 37-year-old woman presented with a 4-month history of right facial pain and a 3-month history of progressive painful swelling beneath the right ear lobe. Examination revealed a soft swelling in the anteroinferior auricular area. The mass was smooth, elastic, mobile and painful on pressure, not fluctuant. The facial nerve function was normal. The cervical lymph node was not palpable. There were no systemic symptoms. The patient was screened for metastases, and no lung or abdomen lesion was detected. Sonography revealed solid echo in right parotid gland and cervical lymph node swelling. A contrast enhanced CT scan revealed a well defined soft tissue mass in right deep parotid lobe (Figure 1).

The standard parotidectomy was performed. A 10 mm × 10 mm superior deep lateral cervical lymph node was resected simultaneously. The rapid intraoperative pathological diagnosis showed the samples from the mass low-grade malignancy and no metastases in the lymph node. Therefore the mass was removed with surgical margin of 1 cm radius around it and preservation of the facial nerve. The surgical margin was microscopically free of tumor. A closed vacuum drainage system was used.

**Figure 2:**
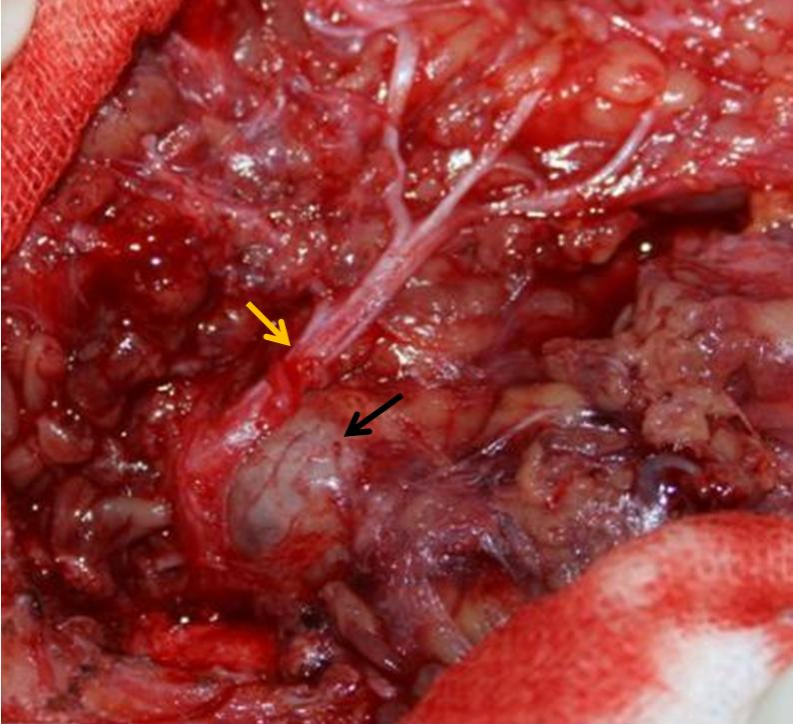
**Relationship between tumor and facial nerve represented during surgery**. The tumor was a grayish-red collagenous mass (black arrow). Main stem of the facial nerve (yellow arrow) was involved.

Macroscopically, the tumor was a grayish-red collagenous mass intermingled with myxoid. Main stem of the facial nerve and the sternocleidomastoid muscle were involved (Figure 2). The excised specimen measured 60 mm × 25 mm × 30 mm.

**Figure 3:**
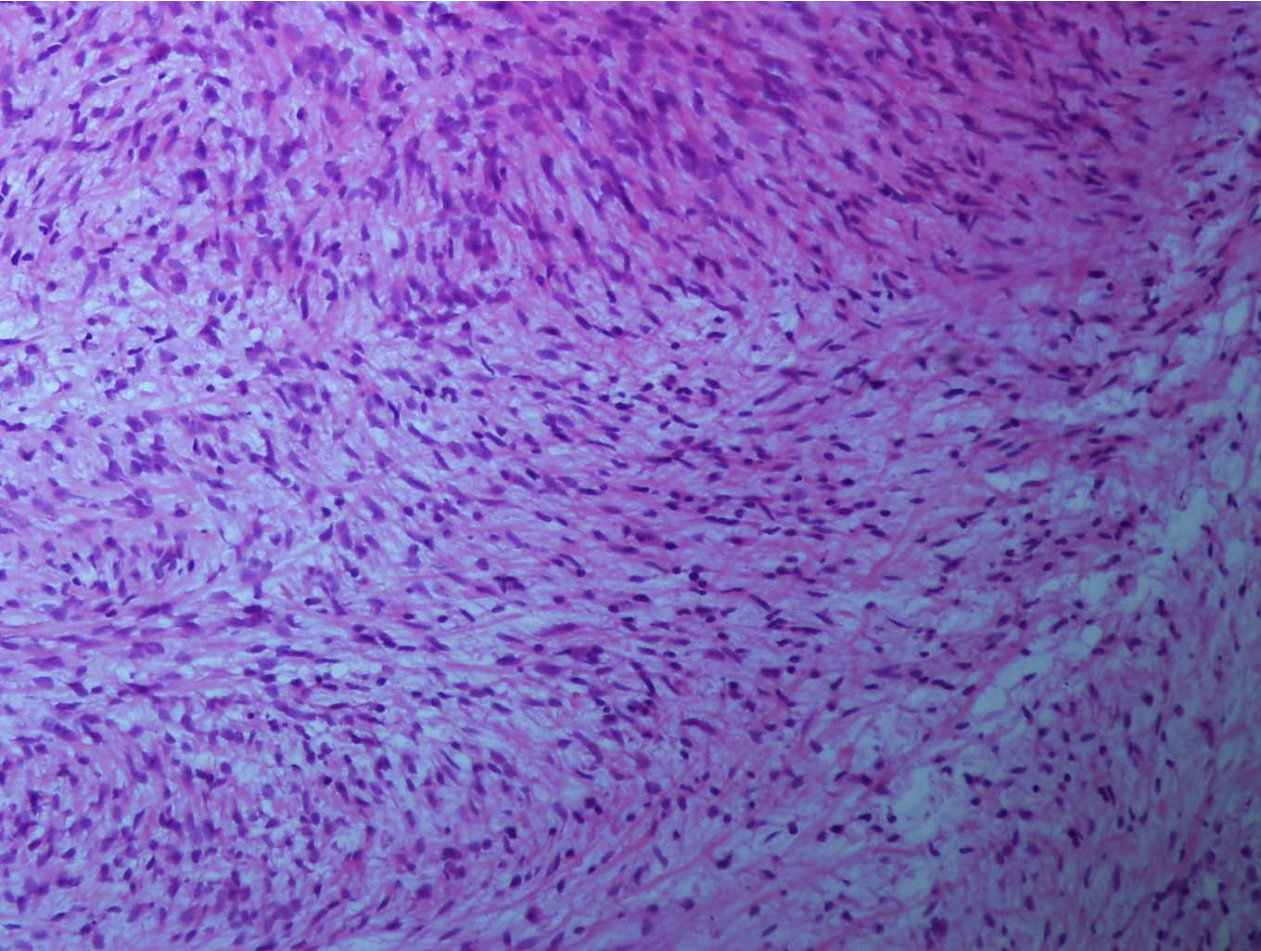
**Photomicrograph of the tumor (H&E; ×100)**. Myxoid areas in association with spindle cells showing a pleomorphic storiform pattern as seen.

Microscopically, the tumor was fairly well circumscribed but not encapsulated perfectly. It consisted of hypocellular to moderately cellular myxoid or fibromyxoid areas. Curvilinear capillaries with perivascular cell condensation were found. Fusiform tumor cells vary from small and bland to enlarged, bizarre, pleomorphic and multinucleated. The cytoplasm of the cells was scant and slightly eosinophilic, and mitoses were infrequent (Figure 3).

**Figure 4:**
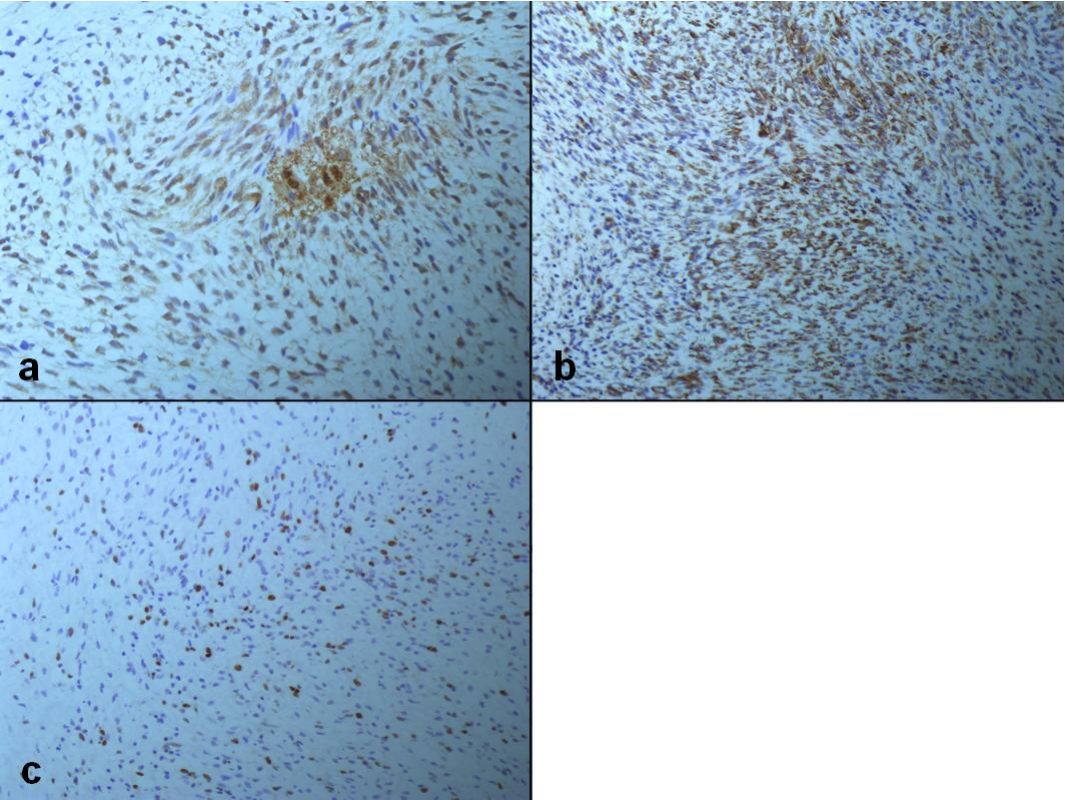
**Positive immunohistochemical findings (immunoperoxidase technique; ×200)**. The tumor cells are positive for ACT (a) and VIM (b). Labeling index of Ki-67 (c) is 35%.

**Figure 5:**
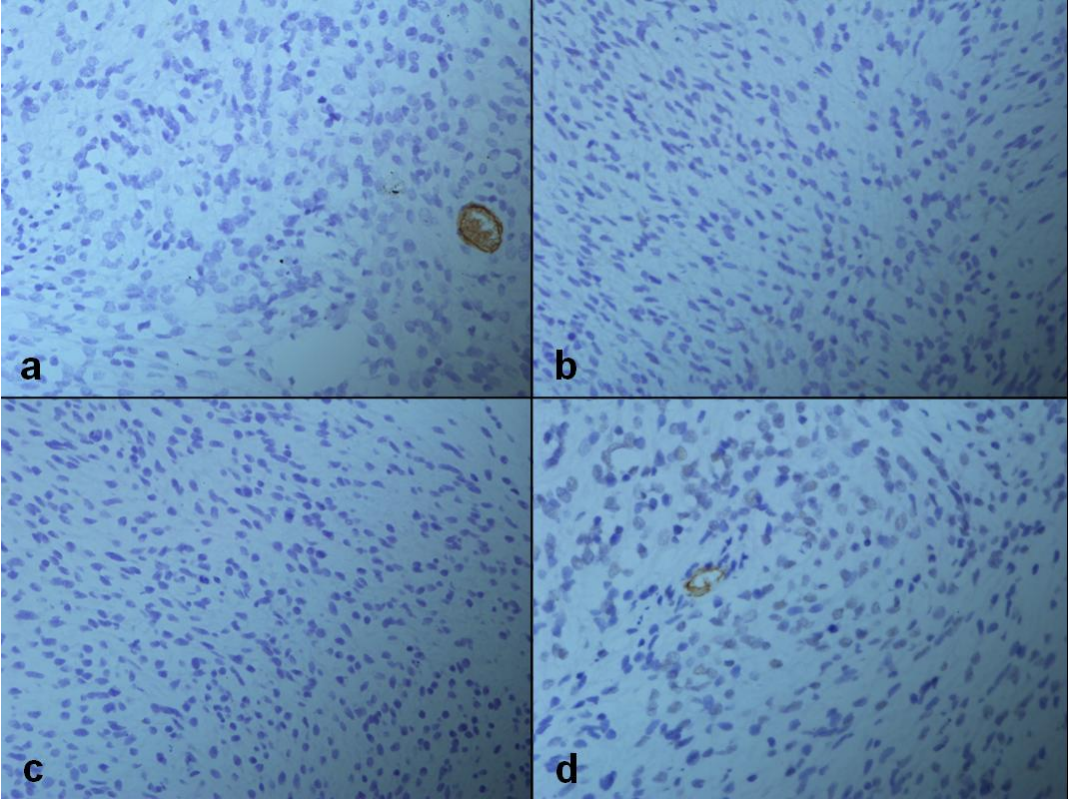
**Negative immunohistochemical findings (immunoperoxidase technique; ×200)**. The tumor cells are negative for CD34 (a), DES (b), MG (c) and SMA (d).

Immunohistochemical studies showed that the tumor cells expressed strong immunoreactivity for vimentin (VIM) and alpha1-antichymotrypsin (α1-ACT). Labeling index of Ki-67 (the number of Ki67 positive tumor cells divided by the sum of Ki67 positive and negative tumor cells) was 35% (Figure 4). Desmin (DES), alpha-smooth muscle actin (α-SMA), beta2-microglobulin (β2-MG) and cluster of differentiation 34 (CD34) assays were negative (Figure 5).

Pathology revealed a T3N0M0 (AJCC 2002) parotid myxofibrosarcoma of low histological grade.

The patient's post-operative clinical course was uneventful. She was discharged on the six post-operative days. The right parotid received the full dose of radiation (68.4 Gy). The patient was free of disease 8 months after surgery.

## Conclusions

Myxofibrosarcoma (MFS), one of soft tissue sarcomas with complex genomic profiles, shows gains and losses of numerous chromosomes or chromosome regions [2]. MFS was described firstly by Angervall as a group of fibroblastic lesions which has cellular distribution, pleomorphism of the nucleus, and mitotic activity that varies from a less cellular lesion with minimal cytologic atypia to a more cellular lesion [3]. As the commonest sarcoma affecting limbs of older patients [1,3-5], high-grade (HG) lesions of MFS were considered to be a myxoid variant of malignant fibrous histiocytomas (MFH) by some investigators [6,7], which was historically classified as a myxoid variant of MFH [8]. However, myxofibrosarcoma is now believed by many authors to be a clinically distinct entity [1,8,9]. They advocated naming it MFS instead of myxoid MFH. This is ascribed to the myxoid nodular appearance, characteristic curvilinear vasculatures, nuclear pleomorphism, histochemical staining, ultrastructural features and its clinicopathologic features [1,4,5,10,11].

According to the study of Antonescu CR and Baren A, basing on microscopic analysis, immunohistostaining by S100 and ultrastructural analysis will help us with the differential diagnosis between myxofibrosarcoma and fibromyxoid sarcoma [12]. Unfortunately, the latter two studies were not performed in this case. While microscopically, the spindled tumor cells, hyperchromatic and irregular nuclei, variable pleomorphism, scattered multinucleated or giant tumor cells and curvilinear-type vessels all verify the tumor is a myxofibrosarcoma instead of a fibromyxoid sarcoma.

Basing on the degree of cellularity, pleomorphism of the nucleus and mitotic activity, myxofibrosarcoma has been divided into three [1,8] or four [1,4,8] grades. Low-grade myxofibrosarcoma has low malignancy with rare distant metastasis and high local recurrence rate (50-60%) [1,4,13], which is similar with that of the high-grade type. Sufficient surgical treatment is the most necessary to suppress local recurrence.

Myxofibrosarcoma is one of the commonest sarcomas of the elder's extremities. It often grows slowly and painlessly with almost equal incidence of men and women. In our case, the patient was just 37-year old, which makes it special. What's more, majority of the tumors are located in the subcutaneous tissue and form multiple gelatinous nodules, which tend to spread in a longitudinal manner. In the head and neck region, myxofibrosarcoma is uncommon. We found some case reports of myxofibrosarcoma in this region, such as in sphenoid sinus, maxillary sinus, hypopharynx, mandible and neck [14-18]. There are also some reports of parotid sarcomas, such as sclerosing rhabdomyosarcoma, synovial sarcoma and interdigitating dendritic cell sarcoma (IDCS), but no myxofibrosarcoma [19-21].

Parotid area swelling should always alert doctors. A unilateral swelling of the parotid implicates a possibility of a rare malignant myxofibrosarcoma. To our knowledge, this is the first case of parotid myxofibrosarcoma. It should be added to the differential diagnosis of diseases of the parotid. We have to recognize this disease and seek adequate treatment for it.

## Competing interests

The authors declare that they have no competing interests.

## Authors' contributions

XL, XC, YC, JHQ, LQ and ZHS were the clinicians who planned the evaluation and procedure. JY was the pathologist who evaluated the specimen. All authors read and approved the final manuscript.

## Consent

Written informed consent was obtained from the patient for publication of this case report and accompanying images. A copy of the written consent is available for review by the Editor-in-Chief of this journal.

## Pre-publication history

The pre-publication history for this paper can be accessed here:

http://www.biomedcentral.com/1471-2407/10/246/prepub
